# Structure-Driven Bioactivity of Chitosan-Agarose-Gelatin Hydrogels Functionalized with Tannic Acid-Cu^2+^/Sr^2+^ Complexes

**DOI:** 10.1021/acsami.6c01091

**Published:** 2026-04-16

**Authors:** Marcin Wekwejt, Pascale Chevallier, Florence Desgagne, Silvia Rodriguez-Fernandez, Elliott Cournoyer, Vanessa P. Houde, Diego Mantovani

**Affiliations:** † Biomaterials Technology Department, Faculty of Mechanical Engineering and Ship Technology, Gdańsk University of Technology, Gdańsk 80-233, Poland; ‡ Laboratory for Biomaterials and Bioengineering, (CRC-Tier I), Department Min-Met-Materials Eng, & Regenerative Medicine, CHU de Québec, Laval University, Québec City G1V 0A6, Canada; § Faculty of Dental Medicine, Oral Ecology 899 Research Group (GREB), 4440 Laval University, 2420 Rue de la Terrasse, Québec City, G1 V 0A6, Canada

**Keywords:** bioactive hydrogel, chitosan, tannic acid, metallic ions, biological performance

## Abstract

Hydrogels represent a versatile platform for regenerative applications; nevertheless, their translation is often limited by insufficient biofunctionality and poorly defined structure–property relationships in multicomponent networks. In chitosan-based hydrogels, performance strongly depends on intrinsic polymer characteristics, requiring precise control of blending and functionalization. Herein, a chitosan-agarose-gelatin blend was functionalized via tannic acid (TA) complexation with Cu^2+^, Sr^2+^, or a dual Cu^2+^/Sr^2+^ system. Chitosan (CS) variants differing in deacetylation degree (DDA), molecular weight (*M*_W_), and biopolymer origin were systematically evaluated to establish design-performance relationships. Effective functionalization occurred only for shrimp-derived chitosan with DDA >90%, confirming the decisive role of polymer chemistry in network formation. Within this design window, *M*_W_ (medium and high) controlled structural organization, degradation kinetics, mechanical performance, and antibacterial efficacy. TA-metal complexes enabled tunable antioxidant and antibacterial responses, with TA-Cu^2+^/Sr^2+^ combined with high-MW chitosan yielding the most balanced multifunctional profile. This formulation preserved a porous architecture, exhibited controlled biodegradation (∼4.5% per month), maintained structural stability over one month, and reached compressive strength and modulus of ∼25.5 kPa and ∼32.6 kPa, respectively. Strong antioxidant capacity and broad-spectrum antibacterial effects were observed as well. Importantly, despite the incorporation of Sr^2+^ for its reported osteoconductive potential, no osteogenic stimulation was observed under the tested conditions. This finding indicates that further optimization of ion ratios and/or TA concentration is required to achieve clinically relevant multibiofunctional hydrogels for bone-related applications.

## Graphical abstract

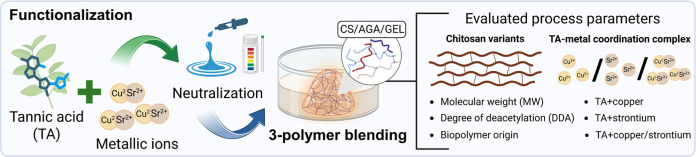

## 1 Introduction

Tissue regeneration plays a pivotal role in the recovery from traumatic injuries and degenerative diseases, with bone repair being particularly demanding in critical-size defects, complex fractures, and osteoporosis. Due to its structural complexity and limited self-healing capacity, bone regeneration remains a significant challenge in regenerative medicine.^1^ Autologous bone grafts are considered the gold standard due to their excellent histocompatibility and low risk of disease transmission. However, their application is limited by complications at the donor site, restricted availability, and the need for additional surgery, which increases the risk of adverse outcomes.^2^ To overcome these limitations, the development of biomaterials that can support and guide bone tissue regeneration has gained increasing attention. To be effective, materials should mimic key features of the extracellular matrix (ECM), promoting cell adhesion, proliferation, and differentiation. They should also be noncytotoxic, nonimmunogenic, biodegradable, and mechanically stable, and present an interconnected porous architecture that enables vascularization, nutrient exchange, and integration with the host tissue.^3,4^

Hydrogels are among the most promising candidates for such materials. Indeed, three-dimensional networks, formed through physical or chemical cross-linking or polymerization, can absorb large amounts of water without compromising their structural integrity.^5^ Their high water content, porosity, soft consistency, biocompatibility, and nontoxicity enable them to mimic the extracellular environment of various tissues.^5,6^ Furthermore, their physicochemical properties can be precisely tuned to meet specific biomedical requirements.^7^ However, despite these advantages, the rational design of multicomponent hydrogels that integrate structural stability with predictable multifunctional bioactivity remains insufficiently defined.^6–8^

Biopolymers are widely used in hydrogel formulations owing to their biodegradability and biocompatibility, which are essential for biomedical applications.^9^ Among them, agarose (AGA), gelatin (GEL), and chitosan (CS) offer complementary features. AGA forms mechanically stable gels upon cooling, but lacks cell-binding sites.^5,10^ GEL promotes cell adhesion, yet suffers from limited mechanical strength.^11^ CS, as a cationic polysaccharide, provides inherent antimicrobial, hemostatic, and anti-inflammatory properties, together with pH-responsive behavior.^12,13^ Importantly, the performance of CS-based systems strongly depends on intrinsic parameters such as origin, degree of deacetylation (DDA), molecular weight (MW), processing conditions and supplier.^14^ These factors influence degradation, mechanical performance, and antibacterial efficacy.^12,14,15^ Despite their recognized importance, intrinsic CS characteristics are rarely systematically evaluated within multicomponent hydrogel systems, limiting the ability to establish clear structure–function relationships.

Polymer blending represents a powerful strategy to combine complementary macromolecules and overcome individual limitations.^16^ Several studies have reported CS-AGA-GEL blends prepared as hydrogels and sponges.^17–20^ However, GEL is typically the predominant component, and chemical cross-linkers such as glutaraldehyde are commonly used.^18–20^ To date, only Hashemi et al. have developed a bioink formulation of this blend in which CS serves as the main polymeric component.^21^ Moreover, rational control of CS intrinsic parameters in relation to network and biofunctional performance remains poorly explored. In our previous work, we established a polymer blend composed of 1.5% CS, 2.0% AGA, and 2.0% GEL (w/v), combined in a 2:1:1 ratio. This system was fabricated using a dual-cross-linking strategy based on the physical gelation of AGA and pH-triggered neutralization of CS, thereby avoiding potentially cytotoxic chemical agents.^22^

To further enhance the hydrogel bioactivity, functionalization strategies are often required.^23^ Tannic acid (TA) can be applied either alone or in combination with bioactive metal ions such as Cu^2+^ and Sr^2+^—an approach that continues to be actively investigated.^24,25^ TA is a polyphenol with antioxidant, antibacterial, and cross-linking capabilities,^24–26^ as well as it forms stable complexes with metal ions.^27^ TA coordination with copper ions, known to exert antibacterial effects and promote osteogenic differentiation^28^ and/or strontium ions, which have osteoconductive properties by stimulating preosteoblast and inhibiting osteoclast-mediated bone resorption,^29^ may contribute to the formation of bone tissue. Both ions also promote angiogenesis, a key process for vascularization and successful tissue integration during bone repair.^28,29^

Given the considerable variability in CS source, MW and DDA, this study investigates how these structural parameters govern the performance of CS-AGA-GEL hydrogels functionalized with TA-metal complexes. Four distinct CS types were selected, blended with AGA and GEL, and subsequently functionalized by incubation in TA solutions containing Cu^2+^, Sr^2+^, or dual Cu^2+^/Sr^2+^ systems under previously established conditions. To the best of our knowledge, this is the first systematic evaluation linking CS structural characteristics to the biofunctional performance of TA-metal functionalized hydrogels. The resulting materials were comprehensively characterized in terms of microstructure, biostability, degradation profiles, biomechanical properties, antioxidant capacity, antibacterial activity against *Staphylococcus aureus* and *Escherichia coli*, and direct cytocompatibility with human osteoblasts, including viability, proliferation, and mineralization.

## 2 Materials and Methods

### 2.1 Hydrogel Preparation

Four types of chitosan (CS; 1.5% w/v; CS_I: Cat. No. 351714, Chitolytic, CA; CS_II: Cat. No. 448877, Sigma-Aldrich, USA, CS_III: Cat. No. 95–401132, Chitolytic, CA, CS_IV: Cat. No. GP5053, Glentham Life Science, UK) were individually dissolved in 1.0% (v/v) acetic acid (Anachemia, CA) under magnetic stirring and left overnight to ensure complete solubilization. In parallel, agarose (AGA; 2.0% w/v, J.T.Baker, USA, low electroendosmosis) was dissolved in nanopure water at 85 °C for 1 h, while gelatin (GEL; 2.0% w/v, Sigma-Aldrich, USA, porcine skin-derived, ∼175 g bloom) was solubilized in nanopure water at 50 °C for the same duration. After complete dissolution, the three polymer solutions were combined in a 2:1:1 (CS/AGA/GEL) volumetric ratio at 45 °C and homogenized for 1 h using a magnetic stirrer. The resulting blend solution (denoted as B; BI–IV based on CS type) was poured into Petri dishes, gelled by cooling in an ice bath, and stored overnight at 4 °C. Cylindrical hydrogel specimens were cut to uniform dimensions using a disposable biopsy punch (Integra Miltex, JP) and subjected to postsynthesis functionalization. The specimens were immersed in an aqueous tannic acid (TA; 1.0% w/v; Sigma-Aldrich, USA) solution containing selected metal ions. Three functionalization conditions were employed: copper (Cu; copper­(II) sulfate; Sigma-Aldrich, USA), strontium (Sr; strontium chloride hexa-hydrate; Sigma-Aldrich, USA), and a Cu/Sr combination (50/50%). The molar ratio of TA to metal ions was maintained at 1:1 to promote stable complexation. After functionalization, all specimens were rinsed and immersed in 1 M NaOH for 10 min (to induce neutralization and partial oxidation of TA), followed by thorough washing with phosphate-buffered saline (PBS, 1×; Sigma-Aldrich, USA). The process parameters, including TA concentration, TA-to-ion ratio, incubation time, and neutralization step, were tested and selected in our previous work.^22^ Notably, the developed hydrogels had already been characterized in terms of their physicochemical properties (i.e., FTIR, TGA, XPS), providing the basis for evaluating their multifunctional biological performance. Importantly, hydrogels functionalized with TA alone were excluded from further analysis because they did not exhibit sufficient structural stability. An overview of all hydrogel compositions is provided in Table 1.

**Table 1:** Detailed Composition of the Developed Hydrogels

	chitosan characteristics^b^				
hydrogel system^a^	degree of deacetylation (%)	molecular weight (kDa)	origin	functionalization strategy^c^	designation
B_CSI	96	120	shrimp	-------------------	
				TA + Cu^2+^	_TA+Cu
				TA + Sr^2+^	_TA+Sr
				TA + Cu^2+^/Sr^2+^ (50%/50%)	_TA+Cu/Sr
B_CSII	>75	190–300	shrimp	-------------------	
				TA + Cu^2+^	_TA+Cu
				TA + Sr^2+^	_TA+Sr
				TA + Cu^2+^/Sr^2+^ (50%/50%)	_TA+Cu/Sr
B_CSIII	98	250–300	mushrooms	-------------------	
				TA + Cu^2+^	_TA+Cu
				TA + Sr^2+^	_TA+Sr
				TA + Cu^2+^/Sr^2+^ (50%/50%)	_TA+Cu/Sr
B_CSIV	>90	1500	shrimp	-------------------	
				TA + Cu^2+^	_TA+Cu
				TA + Sr^2+^	_TA+Sr
				TA + Cu^2+^/Sr^2+^ (50%/50%)	_TA+Cu/Sr

a System composition (constant for all groups): 1.5% CS/2.0% AGA/2.0% GEL (2:1:1).

b Based on supplier specifications.

c Applied strategy (constant for all groups): 1 h; 1% TA w/v; metal ions: 1:1 mol vs TA.

Following the initial screening, only two chitosan-based hydrogel groups (B_CSI and B_CSIV) were selected for further analysis and are presented as the main results. Further, for in vitro cytocompatibility studies with human primary osteoblasts, only TA+Cu/Sr functionalization was employed.

### 2.2 Microstructure

To investigate the internal architecture of the developed hydrogels, specimens were initially freeze-dried to preserve their microstructure (−55 °C, 0.05 mbar; Lyovapor L-200, BUCHI, USA) for 48 h. Once dehydrated, specimens (*n* = 2) were fractured under liquid nitrogen using a scalpel to obtain cross-sections, minimizing deformation and artifacts. The microstructure was analyzed by scanning electron microscopy (SEM). Prior to imaging, each specimen was coated with a thin layer of gold–palladium using a high-vacuum sputter coater Polaron SC500 (Quorum, UK). SEM observations were conducted using a Quanta 250 microscope (FEI; ThermoFisher Scientific, USA) operated at an acceleration voltage of 7.50 kV. For each specimen, images were captured from multiple regions at 50× and 100× magnification. Quantitative assessment of pore size was performed using ImageJ (NIH, USA) and included at least 20 independent measurements per formulation.

### 2.3 Biodegradation and Stability

The in vitro biodegradation behavior of the hydrogels was assessed by quantifying mass loss and monitoring the maintenance of their macroscopic structure during incubation. Disk-shaped specimens (diameter = 8.00 mm and thickness = 3.50 mm; *n* = 4), previously weighed to determine their initial mass (*m*_o_), were incubated in PBS (pH 7.4) at 37 °C. To ensure physiological relevance and prevent ion saturation, the PBS medium was renewed every third day. At predefined time intervals, specimens were retrieved, rinsed briefly with nanopure water, gently wiped with a laboratory wipe, and reweighed (*m*) using a laboratory-scale analytical balance with a resolution of 1.0 mg. The percentage of mass loss, reflecting hydrogel biodegradability, was calculated according to the following eq 1

1 $$degradation\left(\%\right)=\frac{\left(m_{o}-m\right)}{m_{0}}\cdot 100\%$$

The test was discontinued once three out of four replicates within a group exhibited pronounced structural breakdown, indicating loss of functional integrity. Additionally, representative photographs of the hydrogels were taken on day 30 of incubation to qualitatively assess morphological stability and disintegration.

### 2.4 Biomechanical Properties

To assess the biomechanical performance of the hydrogels, uniaxial compression tests were conducted using a mechanical tester (MACH-1, Biomomentum, CA). Disk-shaped specimens (diameter = 18 mm and thickness = 4.2 mm; *n* = 5) were preconditioned in PBS solution for at least 2 h prior to testing. All measurements were performed in a PBS bath within the testing chamber. Compression was applied at a constant deformation rate corresponding to 5% of the specimen height per minute, using a 17 N load cell. The resulting force–displacement data were converted to a stress–strain curve using the integrated system software. The Young’s modulus (*E*_c_) was calculated from the linear region of the curve, excluding the initial toe region. Compressive strength (σ_c_) was defined as the maximum stress prior to fracture; if the material did not fail, the stress at 60% strain was used as a defined endpoint.

### 2.5 Antioxidant Capacity

The antioxidant potential of the hydrogels was evaluated to assess their ability to mitigate oxidative stress relevant to regenerative environments, by measuring their capacity to scavenge DPPH (2,2-diphenyl-1-picrylhydrazyl) free radicals, following a previously reported protocol with slight modifications.^30,31^ Specifically, to obtain the test extract, 100 mg of each hydrogel specimen (*n* = 4; cut into small pieces) was incubated in 400 μL of 1% (v/v) acetic acid (Anachemia, CA) at 37 °C for 2 h. Separately, a fresh DPPH solution was prepared by dissolving 7.89 mg of DPPH in 100 mL of 99.5% ethanol (Greenfield Global, CA) and protected from light during preparation and storage. The solution was used within 4 h to ensure stability. For the antioxidant assay, 200 μL of the hydrogel extract was mixed with 1000 μL of DPPH solution and 800 μL of Tris–HCl Buffer (pH 7.4). The mixture was incubated in the dark at room temperature for 30 min. The final DPPH concentration was 39.45 mg/L. After incubation, the absorbance of each specimen was measured in triplicate at 517 nm using a multiwell plate reader SpectraMax i3x (Molecular Devices, CA). A blank control, composed of 200 μL acetic acid, 1000 μL of ethanol, and 800 μL of Tris–HCl buffer (without hydrogel extract), was used to calibrate the spectrophotometer. The percentage of DPPH radical inhibition was calculated using the following eq 2

2 $$DPPH\;inhibition\left(\%\right)=\frac{\left({Abs}_{DPPH}-{Abs}_{test}\right)}{{Abs}_{DPPH}}\cdot 100\%$$

where Abs_DPPH_ is the absorbance of the DPPH solution alone, and Abs_test_ is the absorbance of the DPPH solution containing the hydrogel extract. As a reference, l-ascorbic acid (Sigma-Aldrich, CA) solutions ranging from 1 μg/mL to 200 μg/mL were used to generate a standard curve.

### 2.6 Antibacterial Activity

The antibacterial performance of the hydrogels was evaluated against two reference bacterial strains: *S. aureus* (ATCC 25923) and *E. coli* (ATCC 25922). Three complementary assays were employed: (1) the disk diffusion method, (2) the bacterial growth inhibition assay, and (3) the bacterial adhesion evaluation. Prior to testing, all hydrogel specimens (disk-shaped; diameter = 8.00 mm and thickness = 3.50 mm) were sterilized by UV irradiation (15 min per side).

#### 2.6.1 Disk Diffusion Assay

To evaluate local bacterial growth inhibition, sterile Trypticase Soy Agar plates (Fisher Scientific, CA) were inoculated with 100 μL of bacterial suspension at a concentration of 1.5 × 10^8^ CFU/mL and spread evenly across the surface. Immediately afterward, hydrogel specimens (*n* = 4) were gently placed directly onto the inoculated agar. Plates were incubated in an inverted position at 37 °C under aerobic conditions for 24 h. Following incubation, the diameter (±0.1 mm) of the inhibition zone surrounding each specimen was measured. Any specimens that shifted position or detached during incubation were excluded from the analysis. As a positive control, an antibiotic disk containing 100 μg/mL of Penicillin–Streptomycin (Invitrogen, USA) was included on each plate.

#### 2.6.2 Bacterial Growth Inhibition Assay

The antibacterial effect in liquid medium was further quantified by monitoring bacterial growth kinetics based on optical density (OD) measurements. A bacterial suspension (1.5 × 10^8^ CFU/mL) was prepared in Trypticase Soy Broth (Fisher Scientific, CA), and 2.0 mL aliquots were transferred to sterile wells of a 6-well plate. Hydrogel specimens (*n* = 4) were added to each well, and plates were incubated statically at 37 °C for 24 h.

At designated time intervals (0, 3, 6, and 24 h), 50 μL of bacterial suspension was collected and transferred into a 96-well plate. The OD values were then recorded at 600 nm using a microplate reader BioRad (Hercules, USA). Bacterial suspensions without hydrogel served as the control group, with their optical density defined as 100%. A decrease in turbidity compared to the control was interpreted as an indication of bacterial growth inhibition.

#### 2.6.3 Bacterial Adhesion to Hydrogel Surface

To complement the quantitative antibacterial assay, selected hydrogel formulations were further examined for bacterial adherence using a direct visualization technique. Following a 24 h incubation in bacterial suspension during the previously described bacterial growth inhibition assay, the specimens (*n* = 4) were gently rinsed with sterile PBS to remove nonadherent bacterial cells. Then, specimens were fixed in 2.5% glutaraldehyde (v/v in PBS; Fisher Scientific, CA) overnight at 4 °C. After fixation, specimens were rinsed again in PBS, dehydrated through a graded ethanol series (30–100%/40 min), and stored at −80 °C until further processing. Subsequent preparation was performed according to the SEM protocol outlined in Section 2.2, including freeze-drying and sputter coating. SEM was used to observe the bacterial adhesion patterns. Images were captured from each tested surface at 2000× and 5000× magnifications. The presence, distribution, and morphology of adhered bacterial cells were qualitatively assessed to compare the surface colonization tendencies of different hydrogel formulations.

### 2.7 In vitro Cell Response

#### 2.7.1 Indirect Osteosarcoma Cytocompatibility

The cytocompatibility of the hydrogel formulations was evaluated using human osteogenic sarcoma cells (Saos-2:89050205; Sigma-Aldrich, USA). Cells were cultured in McCoy’s 5A medium supplemented with l-glutamine (Gibco, CA), 15% Fetal Bovine Serum (FBS; Gibco, CA), and 1% Penicillin–Streptomycin (10,000 U/mL; Gibco, CA). Cultures were incubated at 37 °C in a humidified atmosphere containing 5% CO_2_ until reaching approximately 80% confluence. Cells were then harvested (0.25% trypsin–EDTA, dilution in PBS (1×); Gibco, CA) and seeded into 96-well plates at a density of 18000 cells/well. After 24 h of preincubation, the culture medium was replaced with hydrogel extracts prepared according to ISO 10993–12 guidelines. Specifically, hydrogel specimens (*n* = 4; disk-shaped; diameter = 8 mm and thickness = 4 mm; sterilization as described above) were incubated in complete culture medium at a min. extraction rate of 0.2 g/mL for 24 h under standard cell culture conditions. After exposure to the extracts for an additional 24 h, cell viability was assessed using a resazurin reduction assay (0.1 mg/mL; 1:10 in culture medium; Milipore Sigma, CA), in accordance with ISO 10993-5. Following 2:30 h of incubation with resazurin reagent, fluorescence was measured using a SpectraMax i3x multimode reader (Molecular Devices, USA) at an excitation/emission wavelength of 545/590 nm. Cell viability was expressed relative to the untreated control—cells cultured on standard tissue culture plastic (TCP), which was set as 1.0 (100%).

#### 2.7.2 Direct Osteoblast Cytocompatibility

Selected hydrogel formulations were further evaluated for their ability to support cell proliferation and cytoskeletal organization using a primary human osteoblast cell line (HOB, C-12720; PromoCell, DE). Cells at passages 5–7 were cultured in Dulbecco-Modified Eagle’s Medium (DMEM; Gibco, CA) supplemented with 1% Penicillin–Streptomycin (10,000 U/mL; Gibco, CA) and 10% Fetal Bovine Serum (FBS; Gibco, CA) at 37 °C. Cultures were incubated at 37 °C in a humidified atmosphere containing 5% CO_2_, until reaching approximately 80% confluence. HOB cells were harvested using 0.05% trypsin prepared in PBS (1×; Gibco, CA). Cytocompatibility was assessed using two direct-contact approaches with hydrogel specimens (*n* = 4; disk-shaped, diameter = 8 mm and thickness = 4 mm). In the first setup, cells (12000/well) were seeded onto TCP using a 100 μL drop of suspension, after which one hydrogel disk was placed into each well of a 24-well plate. In the second setup, hydrogels were positioned in nonadherent 24-well plates and cells (15000/material) were seeded directly onto their surface using a 20 μL drop. Following dropwise seeding, cells were allowed to attach for 1 h before adding 2.0 mL of culture medium. Cell proliferation was quantified on days 1, 3, 7, and 14 using the resazurin assay described earlier.

For qualitative assessment after 14 days, specimens from the direct-seeding setup were fixed and stained with Phalloidin-Rhodamine and DAPI (4′,6-diamidino-2-phenylindole) to visualize cytoskeletal architecture and nuclear morphology. Briefly, specimens (*n* = 3) were washed with PBS, fixed with ∼3.7% formaldehyde (Sigma-Aldrich, USA; 2 h, room temperature), and permeabilized with 0.1% Triton X-100 (Sigma-Aldrich, USA; 4 min, room temperature). The actin cytoskeleton was stained by incubating the specimens for 1 h in 0.5× Phalloidin (Abcam, UK; ab235138) diluted in 1% bovine serum albumin (BSA; Sigma-Aldrich, USA) at room temperature. Following PBS washes, nuclei were counterstained with 300 nM DAPI (Sigma-Aldrich, USA) in BSA for 10 min. After final rinsing in PBS, cell morphology was visualized using a confocal laser microscope LSM800 (Carl Zeiss, DE). Moreover, cell quantification was performed using ImageJ software (NIH, USA). For each image (mag. 5×; *n* = 2), four regions of interest (ROI) with an area of 500 μm^2^ were selected. Blue-stained nuclei were counted within each ROI, and the cell number was expressed as mean ± SD.

After 14 days of osteoblast incubation with hydrogels (*n* = 4), ALP activity and DNA quantification were assessed. Cells were lysed through three freeze–thaw cycles, following the manufacturer’s instructions. Briefly, for the ALP assay, enzyme activity was quantified spectrophotometrically using a *p*-nitrophenyl phosphate (*p*NPP) substrate kit (ThermoFisher Scientific, USA). The kinetic reaction was monitored for 30 min at room temperature at 405 nm, after which it was stopped by adding 1 M NaOH. The endpoint absorbance was then measured. ALP activity values were normalized to DNA content, quantified spectrofluorometrically (excitation: 485 nm/emission: 535 nm) using a Quant-iT PicoGreen dsDNA kit (Thermo Fisher Scientific, USA).

### 2.8 Statistics

Statistical data analysis was performed using commercial software (SigmaPlot 14.0, Systat Software, San Jose, CA, USA). The Shapiro–Wilk test was used to assess the normality of the data. All results were calculated as means ± standard deviations (SD) and statistically analyzed using one-way analysis of variance (one-way ANOVA). Multiple comparisons between the control group and the experimental groups were performed using the Bonferroni *t*-test, with a significance level set at *p* < 0.05.

## 3 Results and Discussion

According to our previous study, CS/AGA/GEL hydrogels functionalized with TA combined with metallic ions for 1 h (with applied neutralization) were identified as the most promising among the tested conditions. They exhibited high temporal stability, optimal physicochemical and mechanical properties, while remaining noncytotoxic.^22^ Detailed analyses indicated that these enhanced properties arise primarily from hydrogen bonding and/or covalent interactions involving the amide II and amide III regions formed during the multistep process (Figure 1). Given that key chitosan characteristics, including MW, DDA, and source, are known to influence such interactions, the present study investigates how variations in these characteristics affect functionalization efficiency and the biological performance of the resulting hydrogels.

**Figure 1:**
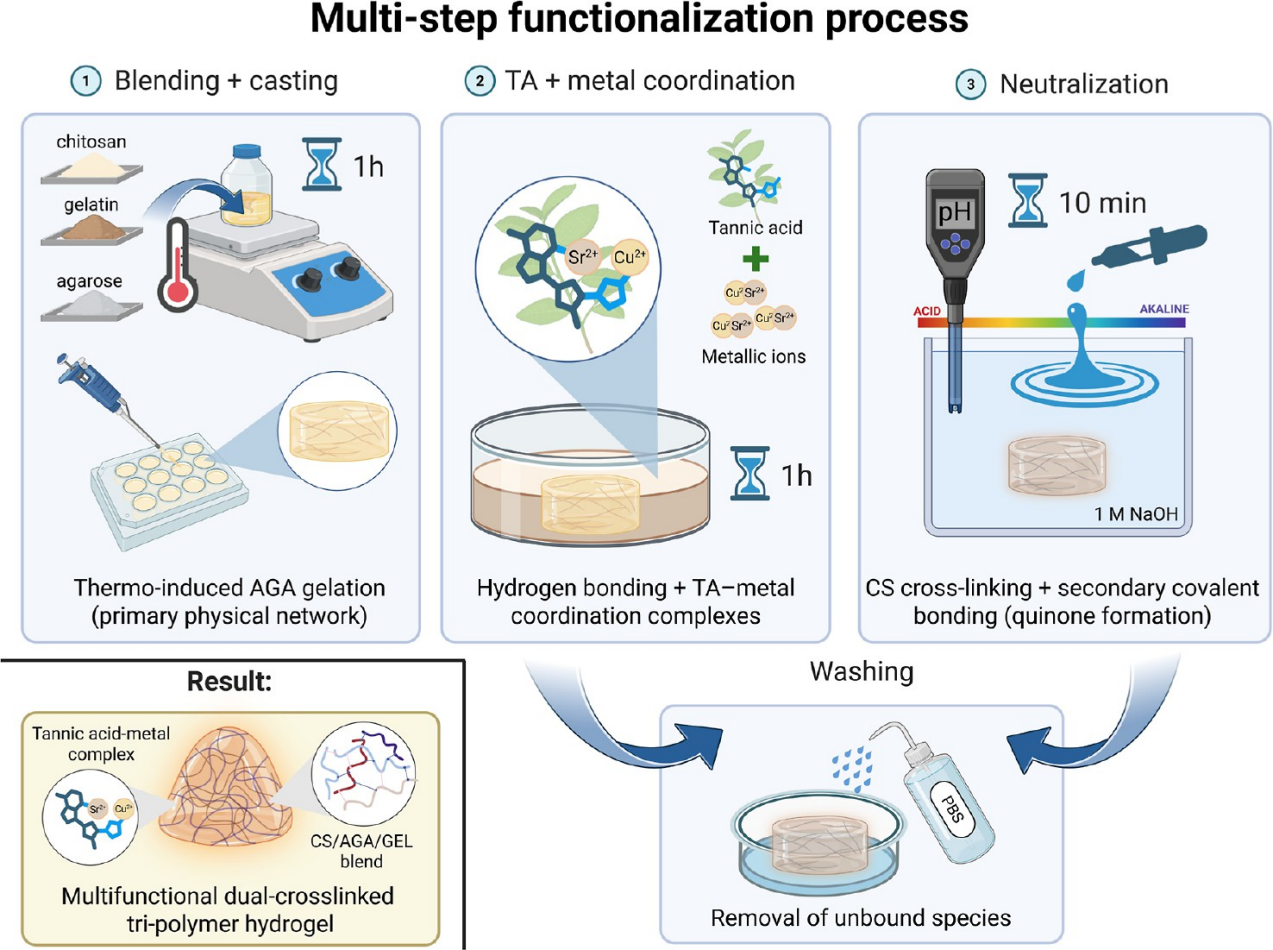
Schematic illustration of the developed multistep functionalization of the chitosan-agarose-gelatin hydrogel and its key interactions.

The results indeed show that the intrinsic features of CS in CS/AGA/GEL hydrogels play a decisive role in determining the final properties after TA and metallic ions functionalization. Among the four evaluated CS types (results in Supporting Information), only two formulations (B_CSI and B_CSIV) were identified as the most promising, likely due to their shrimp origin and high DDA (>90%). For instance, B_CSIII, derived from mushrooms and also characterized by a high DDA (98%), consistently exhibited inferior performance. This behavior may be attributed to its nonuniform chemical structure and the possible presence of residual glucans. These structural features can sterically hinder amino groups, thereby reducing their effective availability for intermolecular interactions.^32,33^ In a similar manner, B_CSII—despite being shrimp-derived, has a lower DDA (>75%) and showed limited suitability for efficient functionalization compared to its counterparts with a higher DDA (CSI and CSIV). This clearly indicates that the density of free amine groups is a key parameter for effective functionalization, as it enables stronger interactions with coordinated TA complexes. The obtained results are consistent with the mechanisms discussed for chitosan in the review by Plota-Pietrzak et al. This work highlights that, beyond the critical role of DDA, the effective availability of amine groups is strongly influenced by polymer chain architecture, purity, and source.^34^ Therefore, only B_CSI and B_CSIV hydrogels were selected for the main investigations, with MW treated as an optimization parameter (medium vs high).

### 3.1 Microstructure Analysis

SEM images (Figures 2 and S1) illustrate the microstructure of the lyophilized hydrogels, which exhibited a characteristic, irregular, sponge-like architecture with pore sizes ranging from approximately 10 to 200 μm. Among the unfunctionalized hydrogels, B_CSIV displayed the largest pore sizes, although its overall microstructure appeared more compact, with noticeably thicker pore walls. Functionalization had varying effects on the architecture, depending on the ions used. In all groups, TA+Cu appeared to markedly alter the microstructure by reducing porosity and disrupting the uniformity of pore distribution. Compared to the unfunctionalized hydrogels, the pores showed reduced interconnectivity and a higher occurrence of closed cavities. This was qualitatively evaluated from SEM cross-sectional images based on pore continuity and the presence of open channels between adjacent pores. In the case of TA+Sr, a notable disturbance of internal porosity was observed only for B_CSI, where pores were less uniformly distributed and exhibited more frequent wall collapse. By contrast, functionalization with TA+Cu/Sr appeared to have the least impact on the microstructure. The pore size distribution, wall thickness, and overall architecture remained comparable to those of the unfunctionalized controls. A general trend toward reduced porosity was also observed.

**Figure 2:**
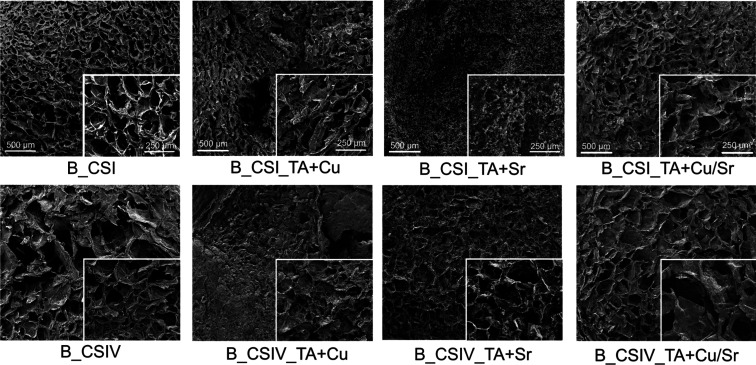
SEM images showing the microstructure of the developed hydrogels after lyophilization (cross-sections; 24 h) at 50× and 100× magnification (images are representative of two analyses).

### 3.2 Biodegradation and Stability Assessment

The biodegradation behavior and stability of the developed hydrogels under simulated physiological conditions are presented in Figures 3 and S2. All tested formulations maintained structural integrity for up to one month, with final mass loss ranging from 4.0% to 26.5%, depending on the composition. Among the unfunctionalized hydrogels, the type of chitosan did not significantly affect the degradation rate. In contrast, following functionalization with TA+Sr, a notably lower degradation was observed for B_CSIV compared to B_CSI (7.7% vs 24.1%, high and medium MW, respectively). Although not supported by statistically significant differences, several visual trends were identified: (1) among unfunctionalized hydrogels, B_CSIV exhibited the lowest degradation, while B_CSI showed the highest; (2) functionalization with TA+Cu was associated with accelerated degradation rates; (3) dual-metal functionalization (TA+Cu/Sr) resulted in the most stable hydrogels overall. Moreover, in the majority of formulations, the most pronounced mass loss occurred within the first 24 h of incubation, particularly for unfunctionalized specimens and those modified with TA+Cu. In contrast, selected formulations containing TA+Sr or TA+Cu/Sr showed their highest degradation in later time intervals, indicating a shift in the dominant degradation phase. This suggests that these functionalizations contribute to a more gradual degradation profile, which may be beneficial in clinical applications requiring extended material stability.

**Figure 3:**
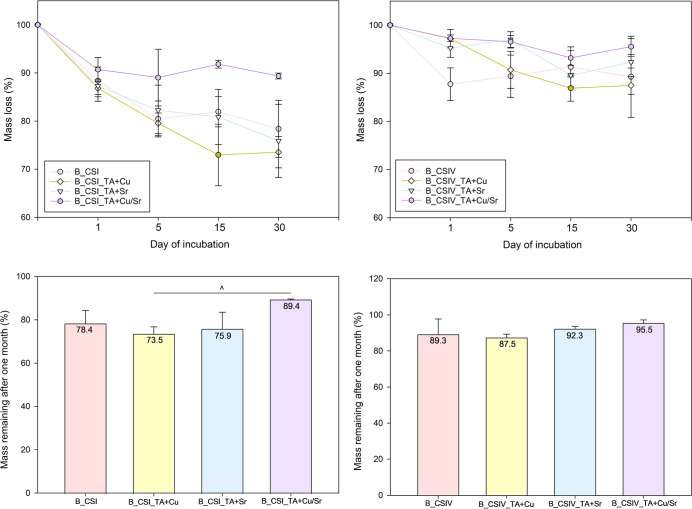
Stability and biodegradation of the developed hydrogels during one month of incubation in PBS solution: degradation profiles and final remaining mass (*n* = 4; data are expressed as mean ± SD * indicates a statistically significant difference compared to the unfunctionalized control (*p* < 0.05); ^ indicates a statistically significant difference between functionalized groups (*p* < 0.05)).

### 3.3 Biomechanical Properties

The results for the biomechanical characterization of the developed hydrogels are summarized in Figure 4 and Table S1. For the Young’s modulus, both chitosan types (with the most pronounced difference observed between CSIII—mushroom and CSIV—shrimp) and the applied functionalization significantly influenced the outcomes. All hydrogels fell within the range of ∼7–33 kPa. In most cases, functionalization with TA+Cu increased the modulus compared to the unmodified counterparts; however, for B_CSIV, a decrease was observed. In contrast, TA+Sr generally reduced the modulus, except for B_CSI, where an increase was recorded. The dual-ion functionalization typically yielded intermediate values between the single-ion modifications. However, in the case of B_CSIV, it induced a synergistic effect, resulting in the highest Young’s modulus across all groups (32.55 kPa).

**Figure 4:**
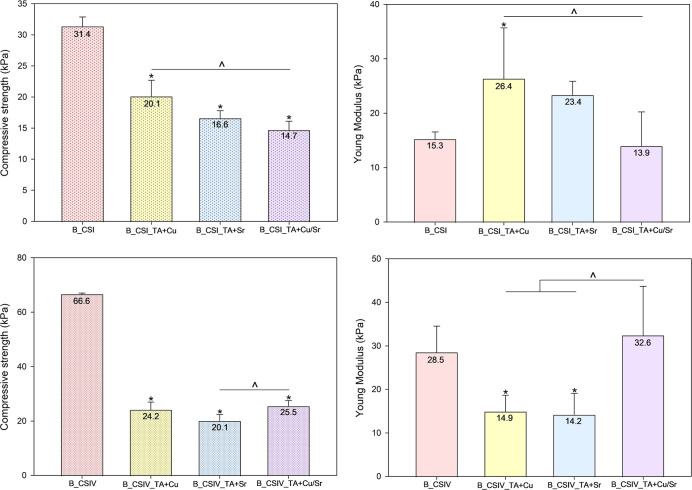
Biomechanical properties of the developed hydrogels tested in PBS solution (*n* = 5; data are expressed as mean ± SD; * indicates a statistically significant difference compared to the unfunctionalized control (*p* < 0.05); ^ indicates a statistically significant difference between functionalized groups (*p* < 0.05)).

Regarding compressive strength, all functionalized hydrogels demonstrated lower values than their unfunctionalized counterparts, ranging from ∼14 to 27 kPa. The strongest unfunctionalized hydrogels were B_CSII (lower DDA) and B_CSIV (exceeding ∼64–66 kPa), whereas the functionalized ones showed reductions of more than 60%. Among the functionalization strategies, TA+Sr consistently led to the lowest compressive strength values, TA+Cu resulted in moderately higher values, and the TA+Cu/Sr variants generally fell between the two, although with noticeable variability. Notably, the B_CSIV_TA+Cu/Sr hydrogel displayed an unusual profile, combining the highest Young’s modulus with only intermediate compressive strength. Overall, the results indicate that while the type of chitosan strongly affects the mechanical performance of the hydrogels, functionalization introduces a trade-off. It can increase stiffness but invariably compromises compressive strength.

### 3.4 Antioxidant Capacity

All functionalized hydrogels exhibited antioxidant capacity ranging from approximately 69% to 78%, depending on the formulation (Figures 5 and S3), which was comparable to the activity of 100 μg/mL ascorbic acid (Figure S4). In contrast, unfunctionalized hydrogels did not demonstrate effective antioxidant activity, although a minor radical scavenging effect was observed for B_CSIII and B_CSIV (∼6–8%; data not shown). No significant influence of chitosan type on the antioxidant performance of the functionalized hydrogels was found. However, a statistically significant enhancement was noted for B_CSIII upon functionalization with TA+Sr compared to TA+Cu (77.9% vs 70.8%), and B_CSIII_TA+Sr also exhibited the highest overall antioxidant capacity. While a general trend toward lower antioxidant activity was observed in hydrogels functionalized with TA+Cu, this trend was not statistically confirmed across all groups.

**Figure 5:**
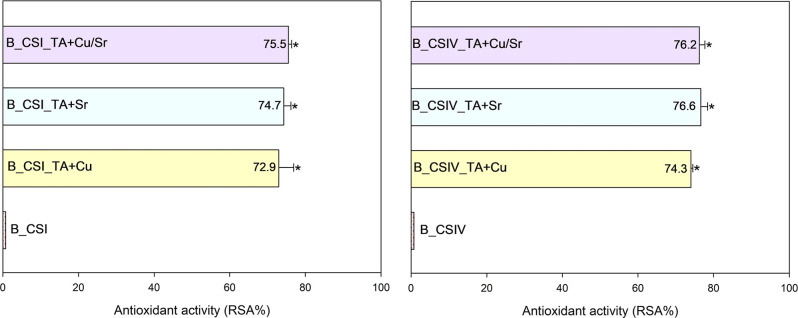
Antioxidant activity of the developed hydrogels expressed as DPPH radical scavenging (%) measured at 517 nm (*n* = 4; data are expressed as mean ± SD; * indicates a statistically significant difference compared to the unfunctionalized control (*p* < 0.05); ^ indicates a statistically significant difference between functionalized groups (*p* < 0.05)).

### 3.5 Antibacterial Activity Against *S. aureus* and *E. coli*

All functionalized hydrogels exhibited antibacterial activity in the disk diffusion method, as indicated by inhibition zones ranging from approximately 1.5 to 3.0 mm (Figures 6B, S5 and S7). This activity was notably lower than that of the positive control—antibiotic disk: 5.79 ± 0.85 mm (Figure S7). No statistically significant differences were observed for either the type of chitosan or the functionalization strategy. However, the highest activity was noted for B_CSII and B_CSI functionalized with TA+Cu/Sr.

**Figure 6:**
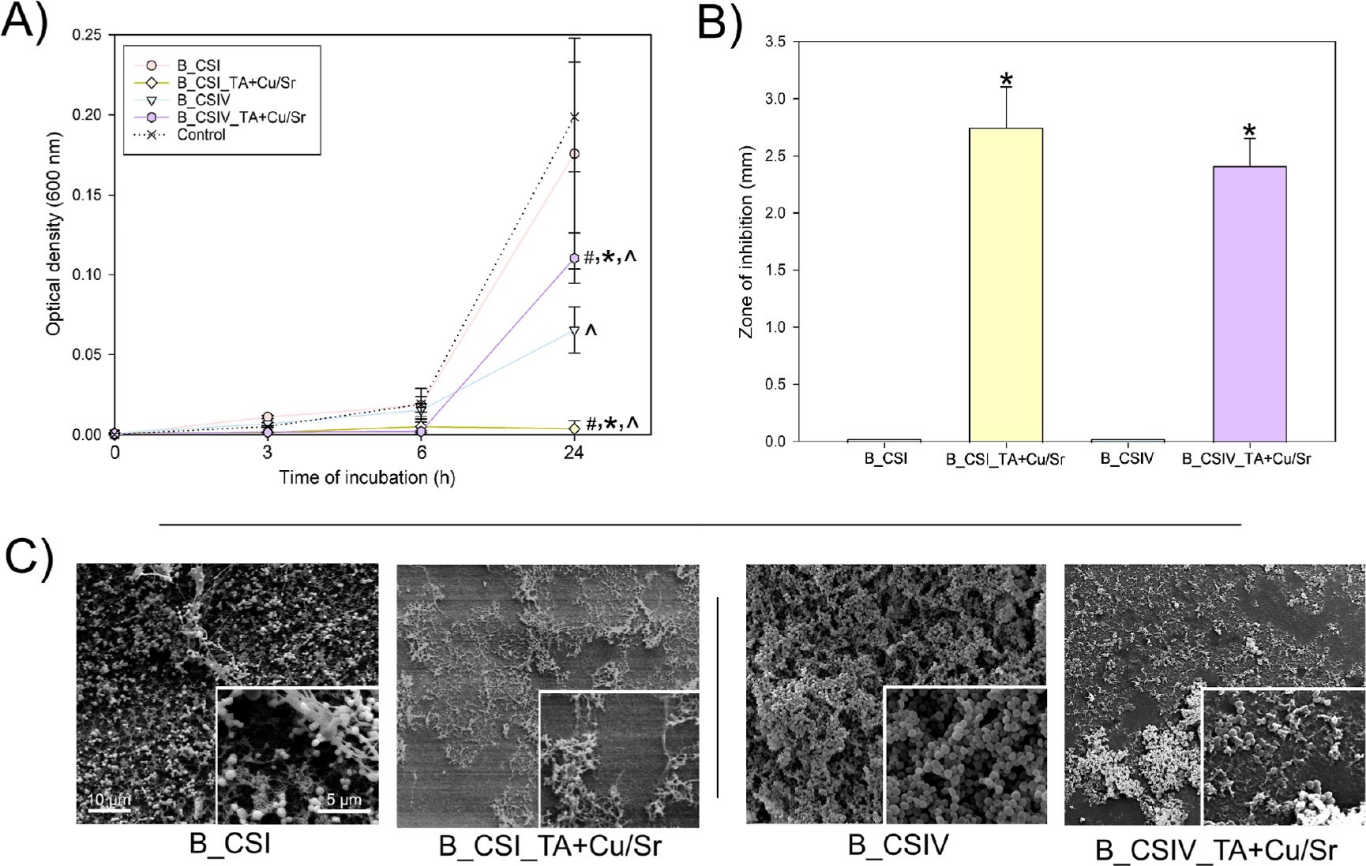
Antibacterial activity of the developed hydrogels against *S. aureus* evaluated in (A) the growth inhibition assay and (B) the disk diffusion assay—presented as inhibition zones after 24 h of incubation (mm); All data are expressed as mean ± SD (*n* = 4; # indicates a statistically significant difference compared to the control (*p* < 0.05); * indicates a statistically significant difference compared to the unfunctionalized hydrogel (*p* < 0.05); ^ indicates a statistically significant difference between the tested functionalized groups (*p* < 0.05)). (C) Bacterial adhesion to the developed hydrogel surfaces after 24 h incubation, examined by SEM after fixation, at 2000× and 5000× magnification.

The bacterial growth inhibition assay indicated that antibacterial performance depended on both the CS type and functionalization, as reflected by differences in growth inhibition over time (Figures 6A and S6). Among the unfunctionalized hydrogels, antibacterial activity was observed exclusively for the CSII-based formulation (lower DDA). Interestingly, this effect was lost following functionalization (except for TA+Cu). The most pronounced antibacterial effects against *S. aureus* were observed for B_CSI functionalized with TA+Sr or TA+Cu/Sr, followed by B_CSIV_TA+Cu, B_CSIII_TA+Cu, and B_CSIV_TA+Cu/Sr. A trend was observed between hydrogels based on different types of chitosan (CSI and CSIV vs CSII and CSIII), as well as between the applied functionalizations.

In SEM images of hydrogel surfaces after 24 h incubation with *S. aureus*, B_CSI displayed a visibly lower number of adhered bacteria (vs B_CSIV), with bacterial clusters occupying less than 50% of the observed surfaces (Figure 6C). Functionalization markedly reduced *S. aureus* adhesion across all selected hydrogel groups. The most pronounced effect was observed for B_CSI_TA+Cu/Sr, where no bacteria were detected on the surface. Interestingly, in the functionalized groups, adhered bacteria were predominantly localized on distinct crystalline structures, which possibly became apparent after PBS washing. Bacterial morphology remained consistent with that of healthy, intact cells, with no significant alterations in shape or size. No evidence of cell deformation, rupture, or shrinkage was detected.

Inhibition zones for *E. coli* ranged from 0.5 to 1.75 mm (Figures 7B, S5 and S7). A trend regarding the influence of both chitosan type (B_CSI and B_CSIV vs B_CSII and B_CSIII) and functionalization (TA+Sr and TA+Cu) was observed. Unfunctionalized hydrogels, except for B_CSI, also showed activity against *E. coli*. However, this response was inconsistent and highly variable (as indicated by a large SD). Reliable antibacterial activity (high zone with low variation) was confirmed only for B_CSI functionalized with TA+Sr or TA+Cu/Sr, and for B_CSIV functionalized with TA+Cu. Compared to the antibiotic control (zone: 3.3 ± 0.3 mm; Figure S7), the activity remained limited.

**Figure 7:**
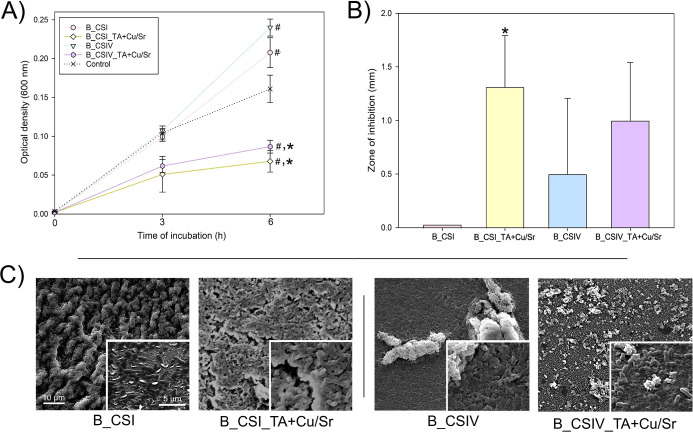
Antibacterial activity of the developed hydrogels against *E. coli* evaluated in (A) the growth inhibition assay and (B) the disk diffusion assay—presented as inhibition zones after 24 h of incubation (mm); All data are expressed as mean ± SD (*n* = 4; # indicates a statistically significant difference compared to the control (*p* < 0.05); * indicates a statistically significant difference compared to the unfunctionalized hydrogel (*p* < 0.05); ^ indicates a statistically significant difference between tested functionalized groups (*p* < 0.05)). (C) Bacterial adhesion to the developed hydrogel surfaces after 24 h incubation, examined by SEM after fixation, at 2000× and 5000× magnification.

No effect on *E. coli* growth inhibition was observed in the assay (Figures 7A and S6), despite visual trends in the data (except for functionalization with TA+Cu). However, a comparable bacterial growth delay was noted for B_CSI, B_CSII, and B_CSIII functionalized with TA+Sr, as well as B_CSI and B_CSIV functionalized with TA+Cu/Sr.

Adhesion of bacteria (Figure 7C) showed that, for the unfunctionalized hydrogels, both formulations exhibited a high density of adhered *E. coli*, forming a continuous biofilm-like layer that covered nearly 90% of the visible surface area. A noticeable reduction in bacterial presence was observed only for B_CSIV_TA+Cu/Sr, where bacterial coverage was reduced to small, isolated clusters. Further, other functionalized groups did not show a substantial difference in adhesion. Bacterial morphology remained similar to that of the healthy ones, with no evidence of deformation, rupture, or shrinkage.

The antibacterial results for the developed hydrogels showed no MW-dependent differences in the disk diffusion assay. However, bacterial growth inhibition and adhesion assays revealed distinct trends (Figures 6 and 7). Functionalized B_CSI showed superior activity against *S. aureus*, effectively limiting both planktonic bacterial growth in the surrounding medium (suggesting a release-mediated effect) and concomitantly reducing bacterial adhesion. For *E. coli*, bacterial proliferation in the suspension was comparably reduced across groups, whereas only B_CSIV strongly inhibited bacterial adhesion. The well-documented antibacterial effects of CS appear to be attenuated within the ternary hydrogel network. Generally, the presence of Cu^2+^ should introduce an antibacterial pathway, involving membrane disruption, interference with intracellular metabolic processes, ROS generation, and interaction with nucleic acids and/or proteins.^35^ However, given the strong antioxidant properties of the developed hydrogels (Figures 5 and S3), the ROS-driven antibacterial effects are unlikely to be apparent under the applied conditions. This may explain the lower efficacy observed with TA+Cu alone. Conversely, several studies have reported the high antibacterial potency of TA+Cu complexes,^36^ and even an enhanced antibacterial effect compared to the individual components, as demonstrated by Chevallier et al.^37^ On the other hand, all assays were conducted over a relatively short time frame. Cu^2+^ ions may require a longer time to be released from the TA complexes at concentrations sufficient to enhance antibacterial action. For example, Zhao et al. reported a continuous and gradual release of Cu^2+^ over 72 h, with the most pronounced increase occurring after ∼10 h, in oxidized hyaluronic acid/carboxymethyl CS hydrogels.^36^ Furthermore, TA+Sr functionalization showed pronounced antibacterial effects against *S. aureus*. Although strontium ions have been reported to exhibit mild antibacterial effects in selected biomaterials, their efficacy is highly dose-dependent.^38^ In TA-based systems, antibacterial performance is therefore likely dominated by TA, with Sr^2+^ providing an auxiliary contribution, as previously demonstrated in Sr-TA-modified nanoparticles incorporated into polymeric dressings.^39^

### 3.6 Indirect In Vitro Osteosarcoma Cytocompatibility

All tested hydrogels demonstrated cytocompatibility with human osteogenic sarcoma cells, as cell viability remained above 70% relative to the TCP control (Figure S8). However, two unfunctionalized hydrogels: B_CSI and B_CSIII (mushroom) exhibited reduced cell viability by approximately 13% and 23%, respectively. Moreover, their viability was significantly lower than that of the other unmodified B_CS hydrogels. For B_CSI, a statistically significant improvement in viability was observed after functionalization with TA+Sr, while B_CSIII also showed a marked increase in cell viability following all applied functionalizations. Notably, the highest cell viability values were recorded for B_CSII and B_CSIV hydrogels functionalized with TA+Cu or TA+Cu/Sr, as well as for B_CSI functionalized with TA+Sr.

### 3.7 Direct In Vitro Osteoblast Cytocompatibility

Among the 16 tested hydrogel variants, the B_CSI_TA+Cu/Sr and B_CSIV_TA+Cu/Sr showed the most favorable combination of microstructure, mechanical integrity, antioxidant capacity and broad-spectrum antibacterial activity. These formulations (and their corresponding controls) were therefore selected as the most suitable candidates for in-depth biological evaluation with human primary osteoblasts. While Saos-2 osteosarcoma cells were employed as a stable and reproducible osteoblast-like screening model, primary osteoblasts more closely represent physiological bone-forming cells and provide higher translational relevance.^40^

Similar to the osteosarcoma model (Figure S8), none of the hydrogels induced cytotoxic effects after 24 h of exposure (Figure 8A). Clear differences emerged in proliferation assays (Figure 8B,C). After 3 days, a measurable reduction in metabolic activity was observed compared with the corresponding controls, ranging from ∼4–20% for cells cultured on TCP and ∼26–67% for cells seeded directly on hydrogel surfaces. This reduction was most pronounced for functionalized hydrogels, particularly for the B_CSI group. Between days 7 and 14, metabolic activity increased across all specimens. However, functionalized hydrogels consistently showed lower proliferation rates relative to their unfunctionalized counterparts. Functionalization also resulted in decreased ALP activity (Figure 8D).

**Figure 8:**
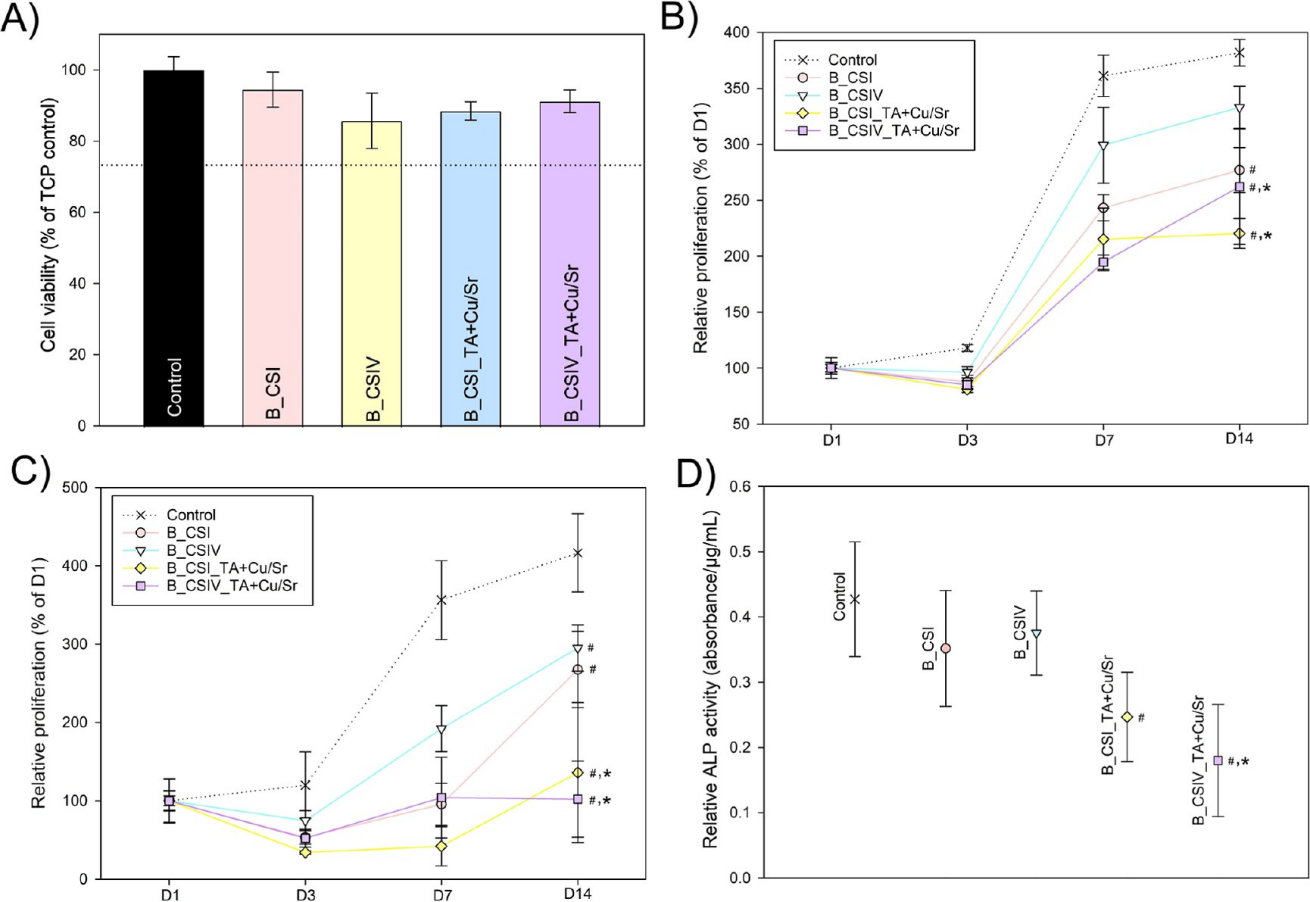
In vitro cytocompatibility of the developed hydrogels evaluated using human primary osteoblasts: (A) direct cytotoxicity (Resazurin assay) after 24 h of cell incubation with materials (the line indictes the noncytotoxic threshold according to ISO 10993-5), (B) cell proliferation after 1, 3, 7, and 14 days of cell incubation with materials (expressed relative to day 1, set as 100%), (C) cell proliferation after 1, 3, 7, and 14 days of cell incubation on the surface of tested materials (expressed relative to day 1, set as 100%) and (D) direct osteogenic differentiation based on alkaline phosphatase (ALP) activity after 14 days (normalized to DNA content (μg/mL)) of cell incubation with materials. All data are expressed as mean ± SD (*n* = 4; # indicates a statistically significant difference compared to the control (*p* < 0.05); * indicates a statistically significant difference compared to the unfunctionalized hydrogel (*p* < 0.05); ^ indicates a statistically significant difference between the tested functionalized groups (*p* < 0.05)).

For the B_CSIV groups, which exhibited a more favorable proliferation rate at day 14, immunofluorescence staining was performed (Figure 9). Both unfunctionalized and functionalized hydrogels supported cell adhesion and preserved osteoblast morphology. Cells displayed the expected osteoblastic morphology, with a spread and elongated appearance, organized actin structures, and uniformly stained nuclei. This confirmed overall healthy cell behavior on both hydrogels, although the functionalized one showed visibly lower cell density. Moreover, cells were distributed uniformly across the surfaces, although a slightly reduced spreading area was noted on the functionalized hydrogels. Quantitative image analysis confirmed a significantly lower cell count on the functionalized material (B_CSIV: 194 ± 26 vs B_CSIV_TA+Cu/Sr: 75 ± 16), corresponding to a ∼2.5-fold reduction and consistent with the proliferation assay results. Those findings suggest that the surface of B_CSIV_TA+Cu/Sr primarily influenced cell number rather than osteoblast adhesion or morphology.

**Figure 9:**
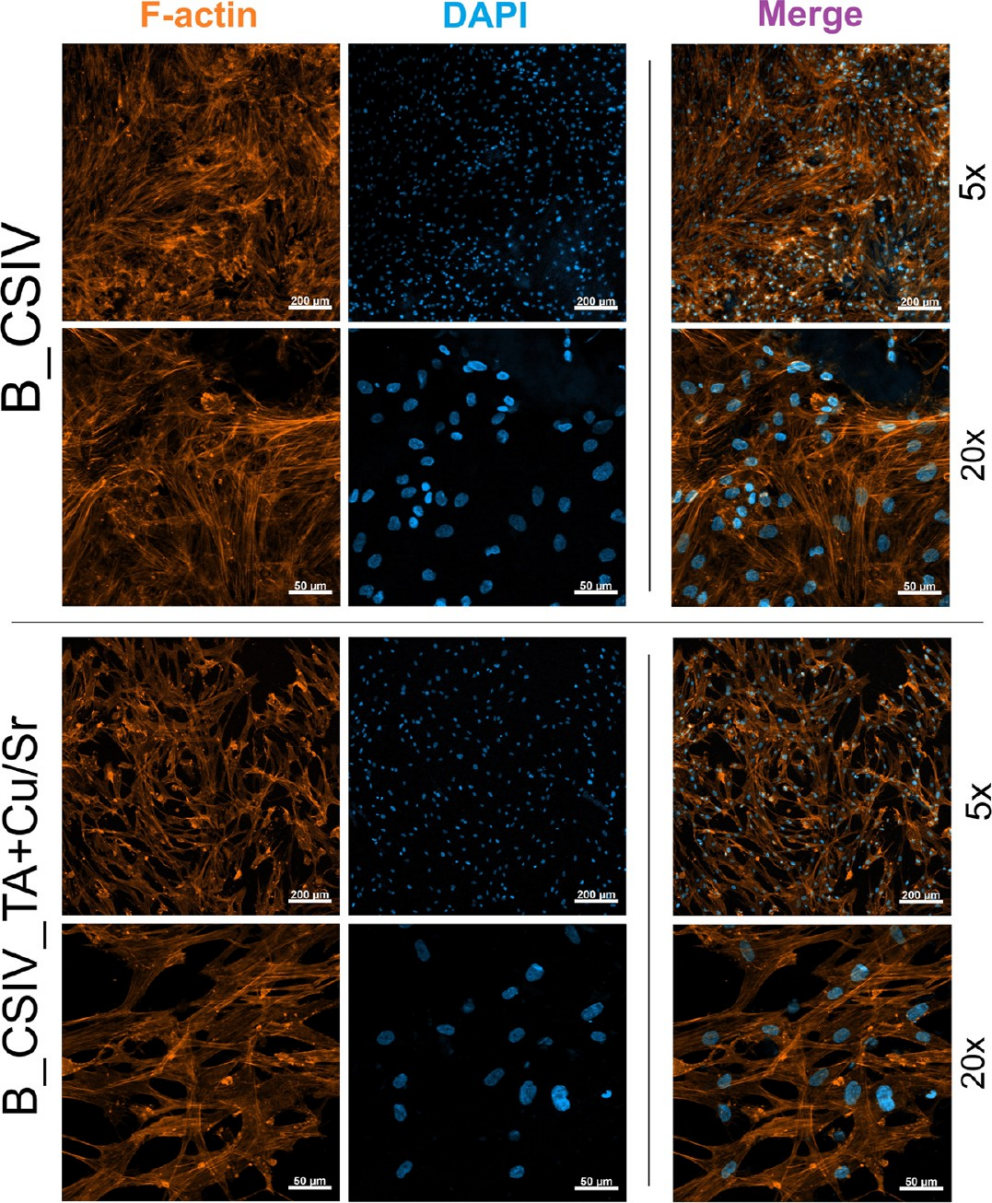
Immunofluorescence-stained images of human primary osteoblasts seeded on the developed hydrogels after 14 days of incubation, showing F-actin (orange; rhodamine–phalloidin) and cell nuclei (blue; DAPI). Images are representative of three analyses.

All hydrogel components (CS/AGA/GEL) are known to be cytocompatible, as also confirmed in our study (Figures S8 and 8A).^41–43^ However, in a CS-based system, it is essential to include an additional neutralization step, as an acidic pH can adversely affect cell behavior.^41^ It is also well established that AGA alone does not support cell adhesion, underscoring the importance of incorporating ECM-related proteins, such as GEL.^42^ In contrast, our proposed hydrogels (both unfunctionalized and functionalized with TA+Cu/Sr) supported cell adhesion (Figures 8C and 9). However, the proliferation rate, tested for B_CSI and B_CSIV, was strongly affected by the applied functionalization, both for cells seeded directly on the material and for those cultured in contact with hydrogels on TCP (Figure 8B,C). The observed decrease in cell metabolic activity, particularly after 3 days, may be related to the release of TA and/or metallic ions. But, as release kinetics were not investigated, the contribution of such mechanisms cannot be directly confirmed. Although TA is widely described as cytocompatible and even pro-osteogenic, some studies have demonstrated that its biological activity is concentration-dependent,^43^ with cytotoxicity effects reported at higher doses.^44^ Similarly, both copper and strontium can exhibit cytotoxic effects when present at excessively high concentrations.^45,46^ Importantly, cell proliferation in contact with the tested hydrogels was primarily slowed rather than completely inhibited, and no evident cytotoxicity was observed, indicating that the cells remained viable. In fact, the immunofluorescence results (Figure 9; B_CSIV_TA+Cu/Sr) clearly confirmed that the osteoblasts retained a healthy morphology on their surface,^47^ despite a ∼2.5-fold decrease in cell number compared to unfunctionalized material. Moreover, the functionalized hydrogels (based on both chitosans) exhibited lower ALP levels compared to the nonfunctionalized controls, indicating no improvement in early osteogenic differentiation. Taken together, the slowed proliferation and reduced ALP activity suggest a mild adverse cellular response to the functionalized hydrogels, consistent with a slightly toxic effect.^48^

### 3.8 Structure–Property Interplay: *M*_W_ vs Functionalization Effects

The results demonstrate that the MW of CS indeed influences the properties of the hydrogels, both in their unfunctionalized state and after functionalization. This is evidenced by differences in microstructure (Figure 2), degradation behavior (Figure 3), mechanical performance (Figure 4), and antibacterial activity against both *S. aureus* and *E. coli* (Figures 7 and 8). In contrast, antioxidant capacity (Figure 5) and indirect cytocompatibility (Figure S8) remained largely unchanged. These effects can be attributed to variations in polymer chain length. Longer chains promoted entanglement and more efficient stress transfer within the blend,^49^ while simultaneously modulating the accessibility of functional groups relevant to antibacterial activity. Although the lower *M*_W_ of CS is generally associated with enhanced antibacterial performance,^50^ this trend was not clearly reflected in the blends, likely because the complex architecture of the multicomponent network can mask this intrinsic property. Consequently, the antibacterial activity of the hydrogels was governed primarily by the functionalization rather than by chitosan *M*_W_ alone.

Among all tested functionalization strategies, only the TA+Cu/Sr system yielded hydrogels with an overall optimal balance of properties. In this case, the microstructure (Figure 2) remained closest to that of the unfunctionalized control, degradation was slowed (significantly for lower-MW CS; Figure 3), and the mechanical performance was only partially affected (Figure 4). Specifically, the compressive strength of B_CSI decreased compared to the single-ion systems, while the Young’s modulus remained similar to the control. Although TA+Cu and TA+Sr functionalization strategies are well documented,^51^ to the best of our knowledge, the combined TA+Cu/Sr system has not been systematically studied. It is worth noting that TA is a multifunctional ligand capable of monodentate, bidentate, and/or bridging coordination modes.^27^ Based on our previous C 1s high-resolution XPS analysis,^22^ Cu^2+^ and Sr,^2^ both individually and in combination, exhibit distinct interaction patterns with TA. These results suggest that the simultaneous presence of both ions may result in more heterogeneous metal-polyphenol coordination. Such interactions are expected to depend strongly on the TA-to-metal ratio, pH, and processing conditions (time and temperature).^52^ This represents a notable gap in the current literature, and our results indicate that the TA+Cu/Sr system provides a distinct and potentially multifunctional response not observed in the single-ion systems. As described previously,^22^ the proposed tripolymer hydrogel undergoes a multistep functionalization process (Figure 1). It is initially stabilized through the physical cross-linking of AGA, followed by subsequent TA treatment (with metallic ions), which predominantly induces hydrogen bonding within the polymer network.^25^ Finally, immersion in 1 M NaOH neutralizes the CS-based matrix and likely promotes additional CS cross-linking^53^ as well as influences functionalization. Under alkaline conditions, phenolic groups of TA deprotonate and oxidize to form quinones—which can participate in further covalent bonding.^54^ As a result, the combined features of this system: tripolymer composition, dual cross-linking, and TA + metal coordination (particularly dual-ion—TA+Cu/Sr), create a material structure that has no direct analogue in existing literature. Within this context, the goal of design was to develop a multifunctional hydrogel offering targeted antibacterial properties while maintaining osteoblast-supportive characteristics.

Importantly, TA+Cu/Sr functionalization achieved a favorable combination of broad-spectrum antibacterial activity (*S. aureus* and *E. coli*) and strong antioxidant capacity. This dual function is advantageous, as radical scavenging helps mitigate oxidative stress and inflammation in the surrounding tissue, thereby creating a more favorable environment for healing and regeneration.^55^ Generally, based on the obtained results, TA appears to be the predominant bioactive component, owing to its well-established antibacterial and antibiofilm activity, particularly against Gram-positive bacteria, such as *S. aureus*.^26,56^ Importantly, TA exerts its antibacterial effects through multiple mechanisms.^57,58^ However, in the current system, the mechanism for chelating metal ions essential to bacterial function may be partially compromised. As TA is already engaged in stable bonding and metal coordination with Cu^2+^/Sr^2+^. A similar phenomenon was reported by Zhang et al., who demonstrated that precoordinated TA exhibits reduced availability of free chelating sites.^59^ However, functionalization with TA alone was not evaluated in this study, as our previous work showed that it compromised the system’s structural stability, leading to disintegration and making it unsuitable for potential biomedical applications.

Finally, although the desired antioxidant and antibacterial performance (both B_CSI and B_CSIV) was achieved, the expected pro-osteogenic effect was not observed. While the present results suggest that the applied functionalization did not promote osteogenic response, they do not allow us to unambiguously identify which component of the functionalization strategy is primarily responsible for this outcome. Importantly, no direct release kinetics of TA or metal ions were evaluated in this study, which limits the mechanistic interpretation of the biological response. The observed effects may be potentially related to several factors, including the relatively high TA concentration (1.0%), the fixed TA-to-metal molar ratio (1:1), or the equal Cu^2+^/Sr^2+^ proportion (50%/50%). However, without quantitative ion release or dose–response data, it remains unclear whether the Sr^2+^ level was suboptimal or the Cu^2+^ content excessive under the tested conditions. Therefore, future work should focus on expanding biological evaluation, detailed release kinetics studies, and/or further systematic optimization of the TA+Cu/Sr functionalization (based on B_CSIV) to enhance the multibiofunctional performance of the hydrogels.

## 4 Conclusions

This study demonstrates that tailoring chitosan/agarose/gelatin hydrogels through precise control of chitosan characteristics (including: degree of deacetylation (DDA), molecular weight (MW), and origin), together with tannic acid (TA)-metal complexation, yields a tunable system with distinct structure–property relationships. Although the hydrogel network also contains other polymers, chitosan (CS) plays an important role. Among the four CS variants initially examined, only those with >90% DDA and shrimp origin enabled efficient TA-based functionalization. Their molecular weight, acting as the primary performance-defining parameter, dictated microstructure organization, degradation kinetics, mechanical strength, and antibacterial activity, while also influencing antioxidant capacity and in vitro cytocompatibility.

Functionalization with TA, simultaneously complexed with Cu^2+^ and Sr^2+^ (1:1 molar ratio with TA; 50%/50%), resulted in the most robust formulation, particularly when combined with high-MW chitosan. This formulation maintained its microstructural architecture during functionalization, exhibited slow and controlled biodegradation (∼4.5% mass loss/month), and achieved mechanical properties suitable for practical handling (compressive strength: ∼25.5 kPa and Young’s modulus: ∼32.6 kPa). This hydrogel (with high-MW) also demonstrated a high antioxidant capacity (∼76.2%) and consistent antibacterial activity, as evidenced by delayed bacterial growth, measurable inhibition zones, and reduced surface colonization by both *S. aureus* and *E. coli*.

Direct osteoblast cultures performed on hydrogels with medium- and high-MW chitosan functionalized with TA and both metallic ions confirmed overall cytocompatibility while revealing reduced cell proliferation and a mild, transient cytotoxic response at day 3, with no enhancement of mineralization. These findings indicate that the current materials may not function as pro-osteogenic scaffolds, as might be expected following Sr^2+^ addition. Instead, they may be better suited as biodegradable antibacterial-antioxidant fillers or implant-adjacent bioactive coatings, where infection control, oxidative stress mitigation, and gradual resorption are required.

Overall, this work established a clear foundation for the rational design of modular chitosan-agarose-gelatin hydrogels based on high-MW chitosan with a high DDA from shrimp origin (as the primary component), functionalized via TA-metal coordination. At the same time, the study identifies the critical limitations of the current systems. Future efforts should focus on refining the ratios of metallic ions and/or TA concentrations to unlock targeted osteogenic functionality and advance these hydrogels toward clinically relevant bone regenerative applications.

## Supplementary Material



## Data Availability

The data that support the findings of this study are available from the corresponding author upon reasonable request.

## Notes

Additional results (including Figures: S1–S8; and table: tS1) for all tested hydrogels: (S1) SEM microstructure, (S2) stability and biodegradation profiles, (tS1) biomechanical properties, (S3) antioxidant activity, (S4) DPPH calibration curve, (S5) antibacterial activity—disk diffusion assay, (S6) antibacterial activity—growth inhibition assay, (S7) representative bacterial inhibition zone images, (S8) cytocompatibility (PDF)

## Abbreviations

AGA: agarose
ANOVA: analysis of variance
B: blend
CS: chitosan
Cu: copper
DDA: degree of deacetylation
DMEM: Dulbecco-Modified Eagle’s Medium
FBS: fetal bovine serum
FTIR: Fourier-transform infrared spectroscopy
GEL: gelatin
HOB: human osteoblast cell line
PBS: phosphate-buffered saline
SEM: scanning electron microscopy
SD: standard deviations
Sr: strontium
TA: tannic acid
TCP: tissue culture plate
