# Supplementary material for: Structure-Driven Bioactivity of Chitosan-Agarose-Gelatin Hydrogels Functionalized with Tannic Acid-Cu2+/Sr2+ Complexes
Source: ACS Appl Mater Interfaces. 2026 Apr 16;18(16):22836–51. doi: 10.1021/acsami.6c01091 (PMC13298822; doi:10.1021/acsami.6c01091)
Supplement: Supplementary file 1 [file am6c01091_si_001.pdf]

Supporting Information

Structure-Driven Bioactivity of Chitosan-Agarose-Gelatin Hydrogels Functionalized with Tannic Acid-Cu^{2+}/Sr^{2+} Complexes

Marcin Wekwejt[*a,b], Pascale Chevallier[b], Florence Desgagne[b], Silvia Rodriguez-Fernandez[b], Elliott Cournoyer[c], Vanessa P. Houde[c], Diego Mantovani[*b]

[a]*Biomaterials Technology Department, Faculty of Mechanical Engineering and Ship Technology, Gdańsk University of Technology, Gdańsk, Poland*
[b]*Laboratory for Biomaterials and Bioengineering, (CRC-Tier I), Dept Min-Met-Materials Eng, & Regenerative Medicine, CHU de Quebec, Laval University, Quebec City, Canada*
[c]*Faculty of Dental Medicine, Oral Ecology Research Group (GREB), Laval University, Canada*
[*] *marcin.wekwejt@pg.edu.pl, diego.mantovani@gmn.ulaval.ca*

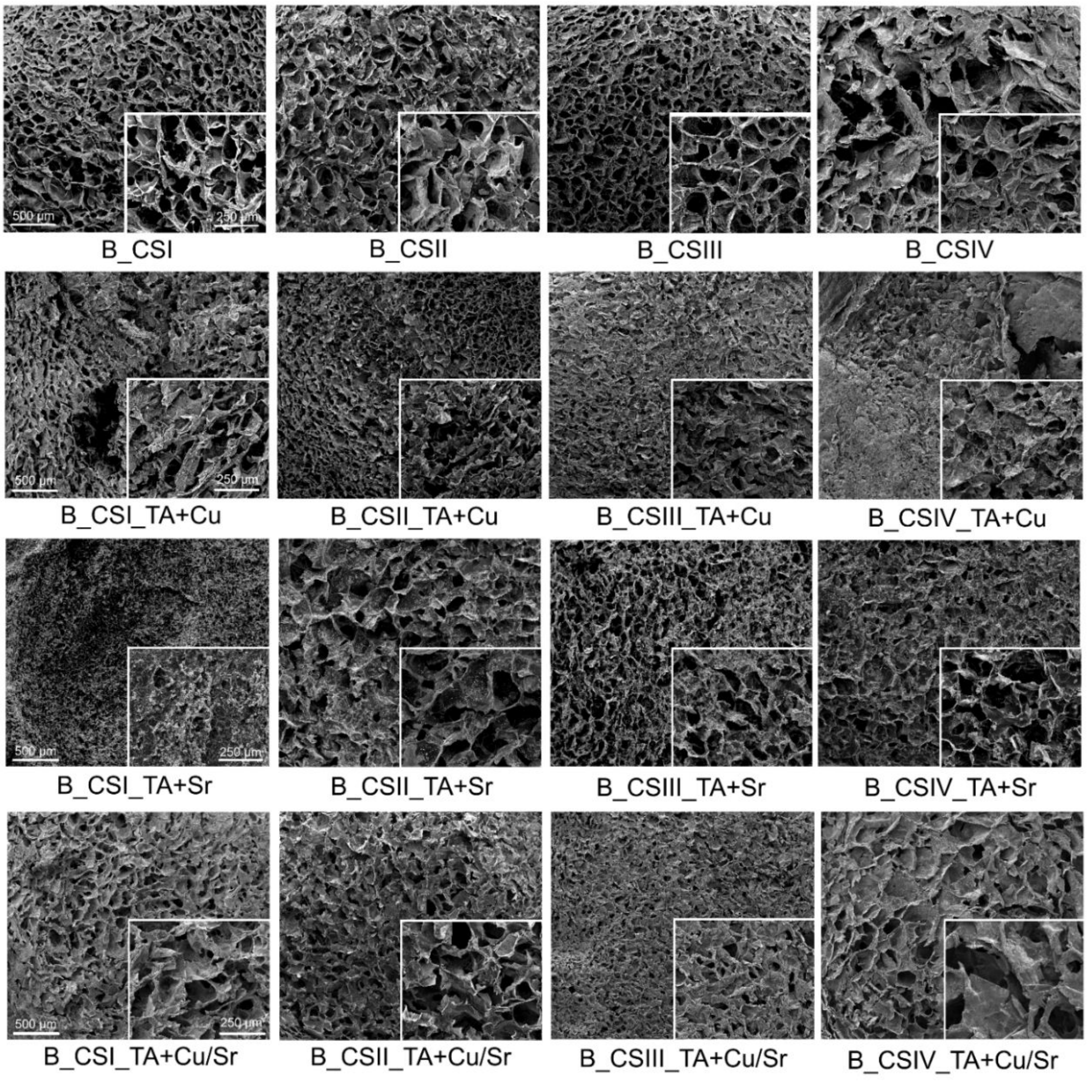

Figure S1. SEM images showing the microstructure of the developed hydrogels after lyophilization (24 h) at 50x and 100x magnification.

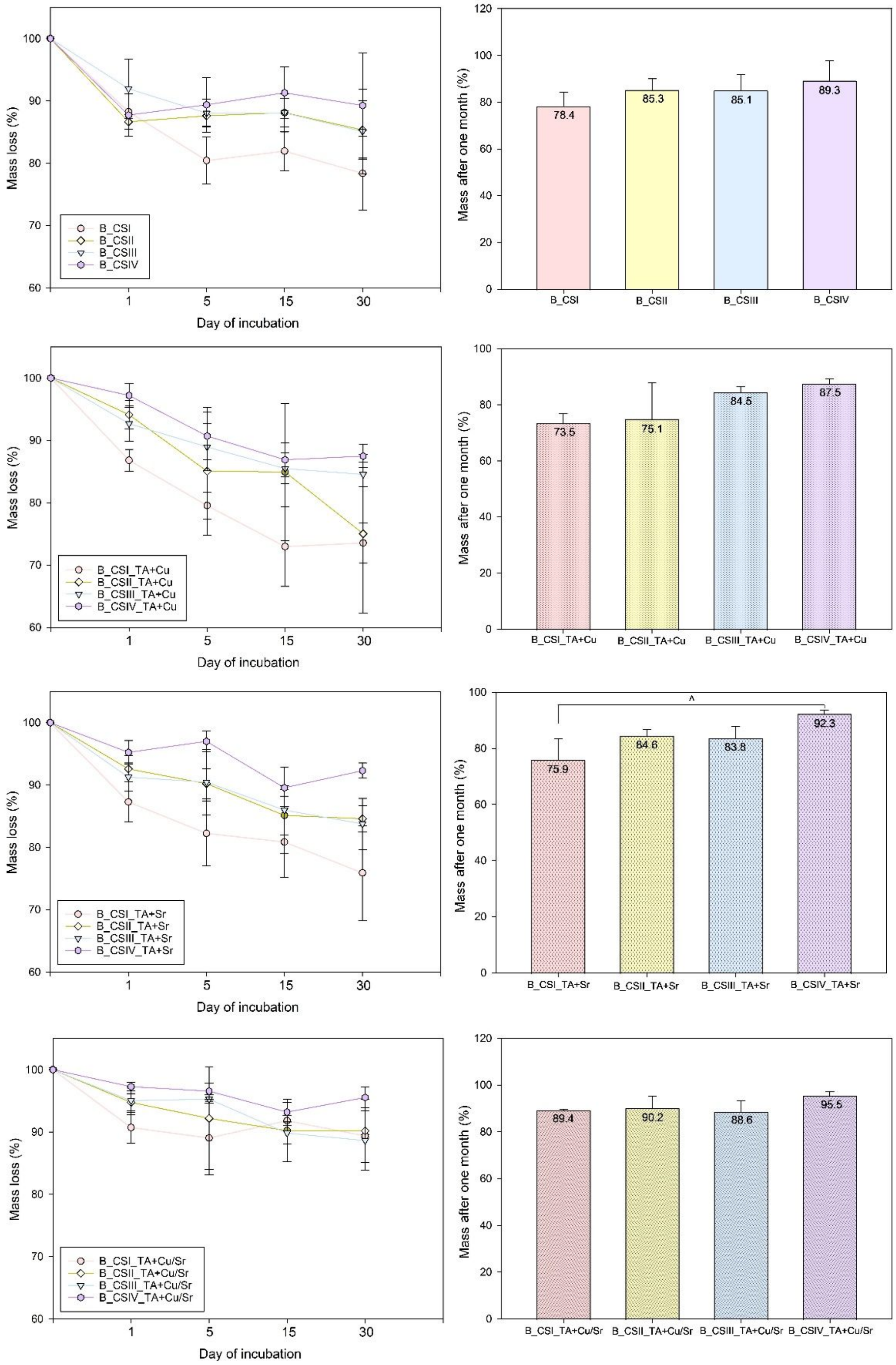

Figure S2. Stability and biodegradation for the developed hydrogels during one month of incubation in PBS solution (n=4; data are expressed as mean ± SD): degradation profile and final mass loss after one month (^ indicates a statistically significant difference compared between types of chitosan (same group of functionalization); $p < 0.05$)).

Table S1. Biomechanical properties of the developed hydrogels tested in PBS solution (n=5; data are expressed as the mean ± SD. ^ indicates a statistically significant difference between types of chitosan (same group of functionalization); $p < 0.05$)).

Hydrogel name	Young Modulus (kPa)	Compressive strength (kPa)
B_CSI	15.26 ± 1.28 $^{\wedge CS:IV}$	31.36 ± 1.52 $^{\wedge CS:II,III,IV}$
B_CSII	16.08 ± 5.79 $^{\wedge CS:III,IV}$	64.61 ± 1.91 $^{\wedge CS:I,III}$
B_CSIII	7.75 ± 1.53 $^{\wedge CS:II,IV}$	55.35 ± 4.32 $^{\wedge CS:I,II,IV}$
B_CSIV	28.53 ± 6.01 $^{\wedge CS:I,II,III}$	66.63 ± 0.40 $^{\wedge CS:I,III}$
B_CSI_TA+Cu	26.36 ± 9.33	20.12 ± 2.57 $^{\wedge CS:III}$
B_CSII_TA+Cu	24.44 ± 4.16	23.79 ± 3.40
B_CSIII_TA+Cu	19.63 ± 13.16	27.08 ± 2.17 $^{\wedge CS:I}$
B_CSIV_TA+Cu	14.92 ± 3.72	24.17 ± 2.81
B_CSI_TA+Sr	23.35 ± 2.51 $^{\wedge CS:II,III,IV}$	16.61 ± 1.20 $^{\wedge CS:IV}$
B_CSII_TA+Sr	9.72 ± 1.95 $^{\wedge CS:I}$	15.41 ± 1.40 $^{\wedge CS:IV}$
B_CSIII_TA+Sr	7.78 ± 2.06 $^{\wedge CS:I,IV}$	15.99 ± 1.52 $^{\wedge CS:IV}$
B_CSIV_TA+Sr	14.21 ± 4.87 $^{\wedge CS:I,III}$	20.14 ± 2.22 $^{\wedge CS:I,II,III}$
B_CSI_TA+Cu/Sr	13.99 ± 6.26 $^{\wedge CS:IV}$	14.69 ± 1.43 $^{\wedge CS:II,IV}$
B_CSII_TA+Cu/Sr	15.83 ± 7.19	21.42 ± 2.29 $^{\wedge CS:I,III,IV}$
B_CSIII_TA+Cu/Sr	15.52 ± 3.44 $^{\wedge CS:IV}$	16.53 ± 1.64 $^{\wedge CS:II,IV}$
B_CSIV_TA+Cu/Sr	32.55 ± 11.24 $^{\wedge CS:I,III}$	25.51 ± 1.96 $^{\wedge CS:I,II,III}$

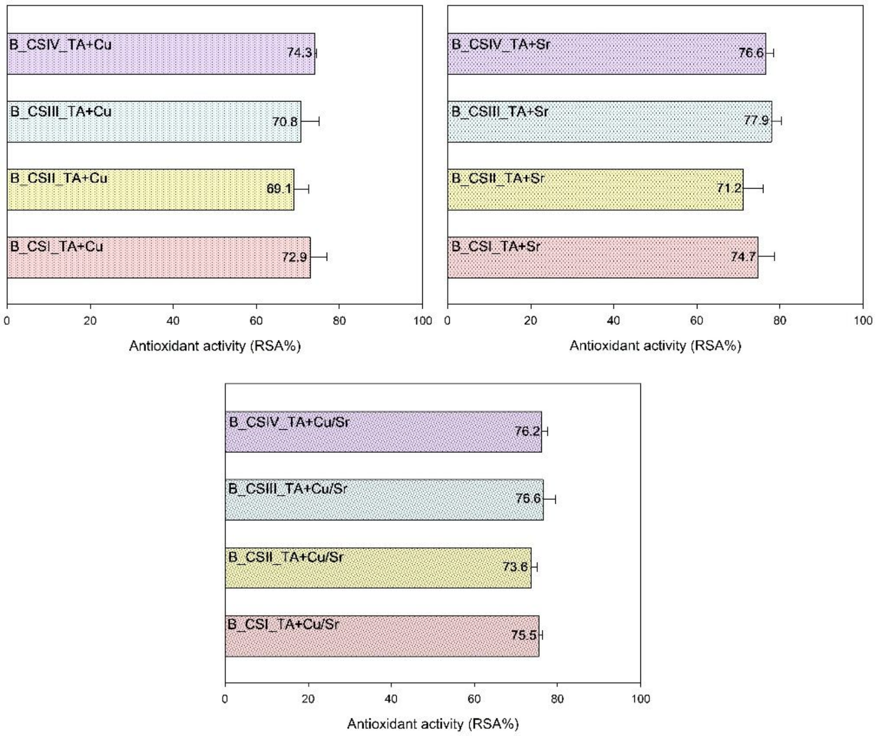

Figure S3. Antioxidant activity of the developed hydrogels expressed as DPPH radical scavenging activity (%) measured at 517 nm. Non-functionalized hydrogels did not exhibit antioxidant capacity (n=4; data are expressed as mean ± SD; ^ indicates a statistically significant difference compared between types of chitosan (same group of functionalization); $p < 0.05$)).

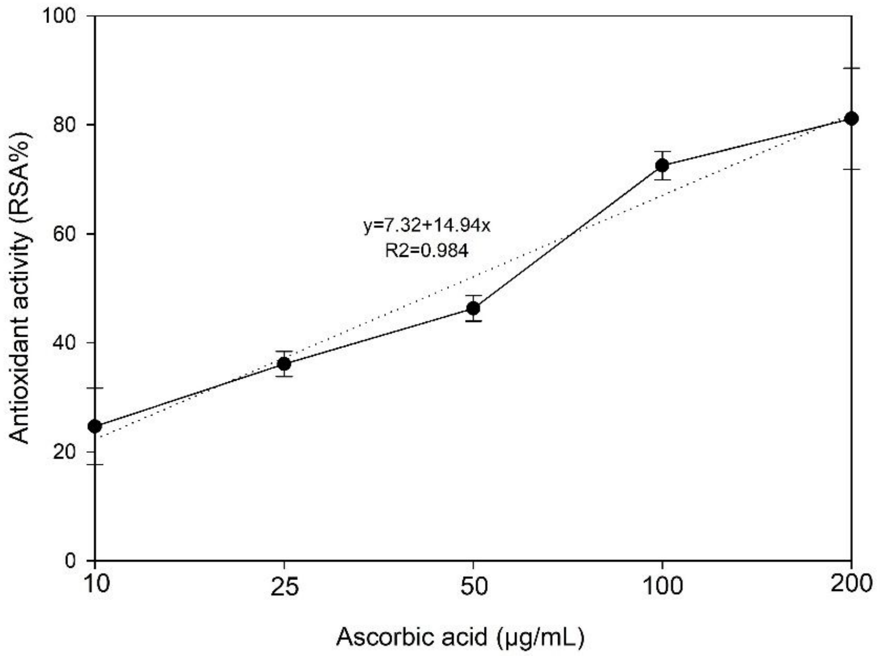

Figure S4. Standard curve for DPPH radical scavenging activity plotted using ascorbic acid as the reference antioxidant (n=3; the dashed line represents the linear fit of the experimental data).

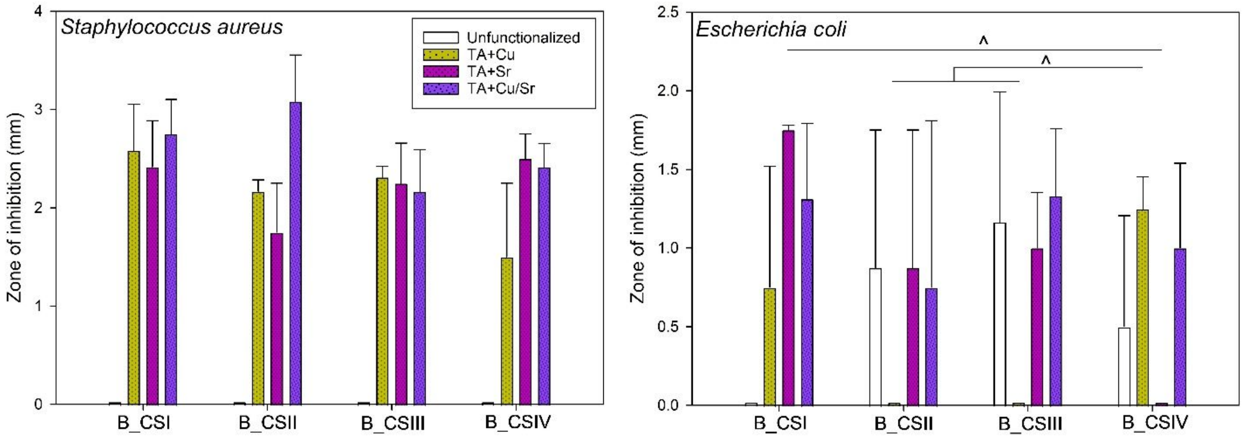

Figure S5. Antibacterial activity of the developed hydrogels against *S. aureus* and *E. coli* evaluated in the disk diffusion assay (as their growth inhibition zones after 24 h of incubation, mm; n=4; data are expressed as mean ± SD; ^ indicates a statistically significant difference compared between types of chitosan (same group of functionalization); $p < 0.05$)).

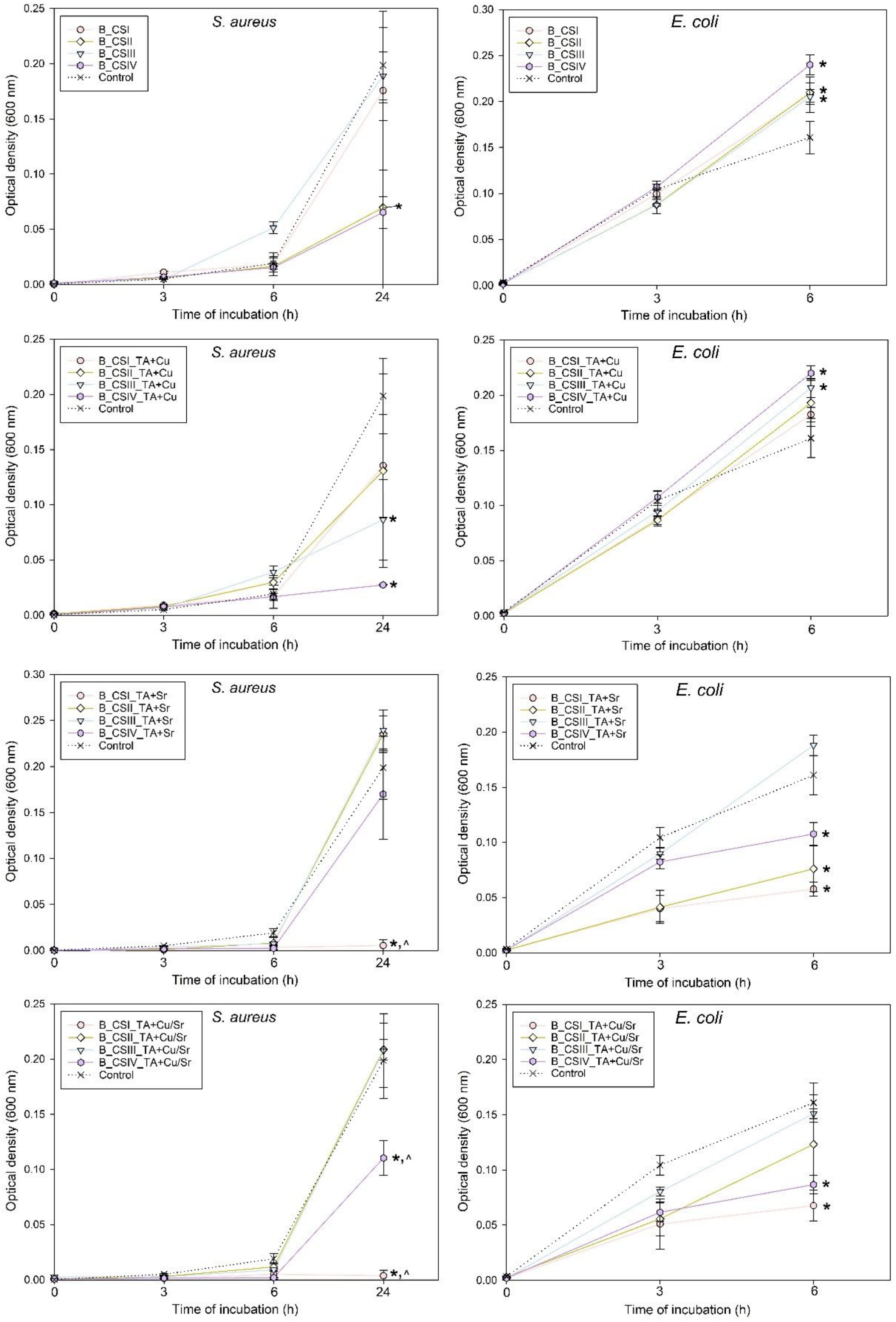

Figure S6. Antibacterial activity of the developed hydrogels against *S. aureus* and *E. coli* evaluated in the bacterial growth inhibition assay (n=4; data are expressed as mean ± SD; * indicates a statistically significant difference compared to the control; $p < 0.05$; ^ indicates a statistically significant difference compared between types of chitosan (same group of functionalization); $p < 0.05$)).

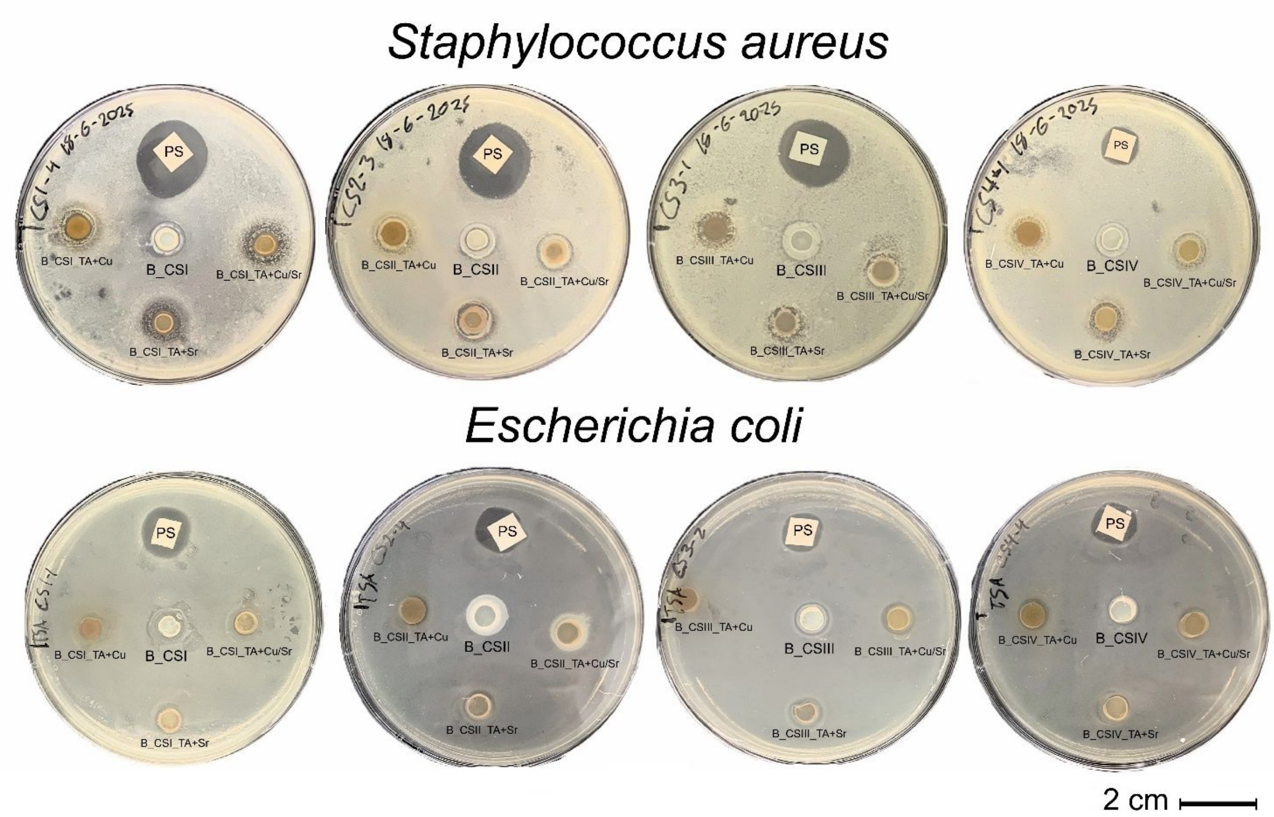

Figure S7. Representative images from the disk diffusion assay after 24 h of incubation (n=4). The positive control (PS) consisted of a commercial antibiotic disk with penicillin-streptomycin.

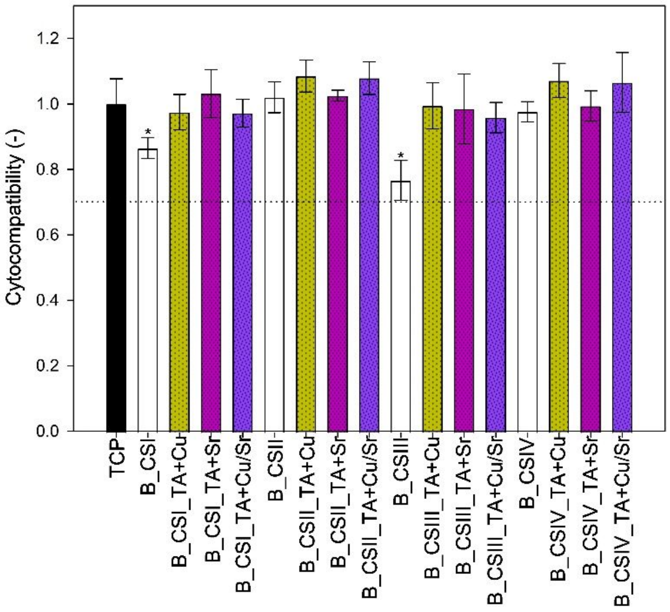

Figure S8. Indirect cytotoxicity test (Resazurin assay) on human osteogenic sarcoma cells after 24h of incubation (n=4; data are expressed as mean ± SD; the line represents the accepted non-cytotoxic limit per ISO10993-5 standard; * indicates a statistically significant difference compared to the control - TCP; $p < 0.05$)).
